# The role of CXCL12 axis in pancreatic cancer: New biomarkers and potential targets

**DOI:** 10.3389/fonc.2023.1154581

**Published:** 2023-03-23

**Authors:** Michela Roberto, Giulia Arrivi, Mattia Alberto Di Civita, Giacomo Barchiesi, Emanuela Pilozzi, Paolo Marchetti, Daniele Santini, Federica Mazzuca, Silverio Tomao

**Affiliations:** ^1^ Oncology Unit (UOC) Oncologia A, Department of Radiological, Oncological and Anathomo-patological Science, Policlinico Umberto I, Sapienza University of Rome, Rome, Italy; ^2^ Oncology Unit, Department of Clinical and Molecular Medicine, Sant’ Andrea University Hospital, Sapienza University of Rome, Rome, Italy; ^3^ Department of Clinical and Molecular Medicine, Anatomia Patologica Unit, Sant’ Andrea University Hospital, Sapienza University of Rome, Rome, Italy; ^4^ Scientific Direction, Istituto Dermopatico dell’Immacolata (IDI-IRCCS), Rome, Italy

**Keywords:** pancreatic ductal adenocarcinoma (PDAC), CXCL12, CXCR4, CXCR7, chemokines, biomarkers, tumor micreoenvironment (TME)

## Abstract

**Introduction:**

Chemokines are small, secreted peptides involved in the mediation of the immune cell recruitment. Chemokines have been implicated in several diseases including autoimmune diseases, viral infections and also played a critical role in the genesis and development of several malignant tumors. CXCL12 is a homeostatic CXC chemokine involved in the process of proliferation, and tumor spread. Pancreatic ductal adenocarcinoma (PDAC) is one of the most aggressive tumors, that is still lacking effective therapies and with a dramatically poor prognosis.

**Method:**

We conducted a scientific literature search on Pubmed and Google Scholar including retrospective, prospective studies and reviews focused on the current research elucidating the emerging role of CXCL12 and its receptors CXCR4 – CXCR7 in the pathogenesis of pancreatic cancer.

**Results:**

Considering the mechanism of immunomodulation of the CXCL12-CXCR4-CXCR7 axis, as well as the potential interaction with the microenvironment in the PDAC, several combined therapeutic approaches have been studied and developed, to overcome the “cold” immunological setting of PDAC, like combining CXCL12 axis inhibitors with anti PD-1/PDL1 drugs.

**Conclusion:**

Understanding the role of this chemokine’s axis in disease initiation and progression may provide the basis for developing new potential biomarkers as well as therapeutic targets for related pancreatic cancers.

## 1 Introduction

Pancreatic ductal adenocarcinoma (PDAC) is one of the most aggressive tumors and the seventh leading cause of cancer-related death worldwide (1). The incidence of PDAC is increasing by 0.5% to 1.0% per year (2) and the 5-years survival rate is 9% (3). Approximately 80% of patients present with advanced disease or distant metastasis at diagnosis, with a very poor prognosis (4). The lack of effective therapeutic opportunities, in part due to the inter- and intratumor heterogeneity, contributes to making PDAC still a deadly malignancy. Multiple signaling pathways involved in pancreatic cancer tumorigenesis - Ras-ERK pathway (5), multiple genes/proteins, such as NOP14 (6), DCLK1 (7), interleukin-22/interleukin-22 receptor (8), FoxQ1 (9), CHIP (10) and microRNAs (11) - were recently found to play an important role in migration and invasion of pancreatic cancer cells. Besides this complex set of pathways, it has recently been highlighted the effect of chemokines in malignant behaviors of cancer cells (12). Chemokines are chemoattracting proteins that binds to and activate their corresponding receptors. There are four families of chemokines, CXC, CC, CX3C, and C. Among them, CXC ligand 12 (CXCL12) of the CXC chemokines family, was previously shown to have important impact on the proliferation and invasion of many types of cancer cells, through its specific receptors CXCR4 (12) CXCR7 (13). In addition, CXCR7 is involved in a broad range of cancer progression processes, such as growth, migration, chemotaxis, adhesion and spreading (14). Specifically, in pancreatic cancer CXCL12-CXCR7 axis promotes migration and invasion of pancreatic cancer cells, through mTOR and Rho/ROCK pathway (15). Tough, it has been hypothesized a prognostic role and a potential therapeutic target for this pathway. In this review, we summarize the current evidences about CXCL12 - CXCR4 - CXCR7 mechanisms of action, especially in PDAC, with the aim to identify new potential biomarkers and therapeutic targets for this aggressive malignancy.

## 2 Methods

A comprehensive literature search in the electronic database of PubMed, and Google Scholar was conducted in January 2023. The literature search was performed using keywords. Retrospective and prospective studies published from January 2020 to December 2022 including the following key words: “CXCL12”, “CXCR4”, “CXCR7”, “axis”, “pancreatic cancer”, “CXCL12 axis in pancreatic cancer”, “CXCL12 and carcinogenesis”, “CXCL12 inhibition”, “CXCL12 and tumor microenvironment” were included in the analysis.

## 3 Critical analysis

### 3.1 Mechanism of action of chemokines: The role in the generation and development of malignant tumors, including PDAC

Chemokines are small, secreted peptides involved in the mediation of immune cell recruitment. They are considered key regulators of development, differentiation and anatomic location of leukocytes and can control cell movement during inflammation and homeostasis (16, 17). Based on the great amount of pathway in which they are involved, chemokines play a critical role in several diseases which comprise autoimmune diseases, viral infections as well as many cancer types (18–24).

**Figure 1:**
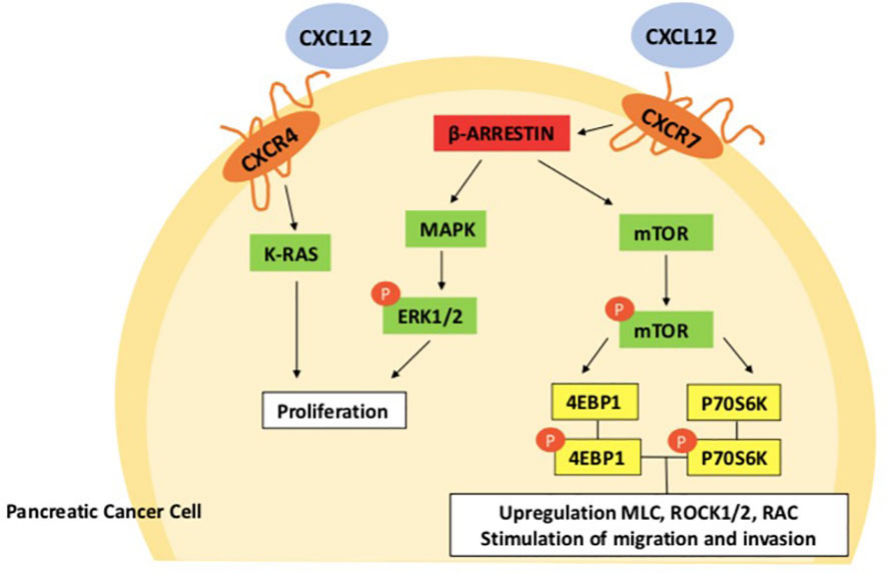
CXCL12-CXCR4-CXCR7 axis in pancreatic cancer cell. After CXCL12 and chemokine (C-X-C motif) receptor 7 (CXCR7) are activated, β-arrestin 2 causes extracellular regulatory protein kinases 1/2 (ERK ½) to become phosphorylated, which promotes the growth of pancreatic cancer cells. By activating the mammalian target of rapamycin (mTOR) signaling pathways, CXCR 7 interacts to stromal cell-derived factor-1 to increase the migration and invasion of pancreatic cancer cells (30). CXCR7, Chemokine (C-X-C motif) receptor 7; SDF-1, Stromal cell-derived factor-1; ERK1/2, Extracellular regulated protein kinases 1/2; mTOR, Mammalian target of rapamycin; Rho/ROCK, Rho/Rho associated coiled-coil forming protein kinase.

Chemokines are divided in four subtypes depending on the position and number of cysteine residues in the N-terminus: C, CC, CXC and CX3C chemokines (25). There are two families of chemokines receptors: conventional (cCKR) and atypical chemokine receptors (ACKR). They exert their functions binding to seven- transmembrane- spanning G protein-coupled cell-surface receptors (26). Ligand-receptor binding induces a conformational change in the receptor which led to intracellular signal transduction and activation of downstream signaling pathways (26–28) (29). In pancreatic cancer, CXCL12 chemokine, released by activated cancer‐associated fibroblasts (CAFs) binds to its two receptors CXCR4 and ACKR3 on the cells surface, activating phospholipase C, MAPK, and PI3K-Akt-mTOR, as well as JAK/STAT pathways, promoting tumor growth and invasion (see **Figure 1**) (31–34).

Some chemokines, specifically the CXC chemokines group, are involved in cancer growth promoting angiogenesis. The presence or lack of the ELR(Glu-Leu-Arg) motif allows CXC chemokines to be classified as angiogenic or angiostatic. More specifically, angiogenesis is stimulated by ELR-positive chemokines (CXCL1, CXCL6, and CXCL8) while it is inhibited by ELR-negative chemokines (CXCL4, CXCL10 an endogenous tumor angiogenesis inhibitor (35) and CXCL14) (36). CXCL12 (SDF-1) is an ELR-negative chemokine but, despite being ELR negative it represents one of the most powerful angiogenesis-promoting chemokines (37).

Focusing on the role of the CXCL12 axis, *in vivo* and *in vitro* studies revealed a relationship between microvessel density and the degree of CXCL12/CXCR4 expression in cancer cells. There are several ways through which CXCL12/CXCR4 axis could control tumor angiogenesis: increasing vascular endothelial growth factor (VEGF) expression in tumor tissue *via* the PI3K/Akt signaling pathway (38); increasing several angiogenesis-related genes in cancer cells (39) and directing endothelial progenitor cells toward the site of neovascularization (40, 41). Indeed, a study showed that human intestinal microvasculature co-expresses CXCR4 and CXCL12 and that CXCL12 promotes chemotaxis, proliferation of endothelial cells and angiogenesis (42). Moreover, CXCL12 positively affects VEGF expression by endothelial cells; consequently, increased VEGF levels up-regulate CXCR4 levels (43).

CXCL12 axis can influence tumor cell biology by direct effects, promoting cancer cell growth, metastasis, and angiogenesis, but also indirectly, recruiting CXCR4/CXCR7-positive cancer cells to CXCL12- expressing organs (44). An experimental study showed that CXCL12 was only weakly expressed in quiescent tissues, but when VEGF expression was experimentally increased, using the conditional genetic switch, the expression of CXCL12 in perivascular fibroblasts and smooth muscle cells grown dramatically. VEGF-dependent mechanisms also led to the induction of CXCL12 in ischemic, inflammatory, and neoplastic tissues (45).

Besides angiogenesis, CXCL12 and its receptor CXCR4 are involved in promoting tumor invasion and metastases in several neoplasm (46–48) including PDAC (15). Some evidence suggested a possible role of CXCL12/CXCR7 on the epithelial-to-mesenchymal transition, a critical step that initiates metastasis in colorectal (49), esophageal (50) and ovarian cancers (51). The relation between metastasizing and angiogenesis was demonstrated by the inhibition of phosphoglycerate kinase (PGK) expression, an angiostatic kinase, by the great expression of CXCL12/CXCR4 on metastasis (52). Moreover, some studies showed that in animal models of breast, renal cell, and non-small cell lung cancer, immunoneutralization of CXCL12 or CXCR4 attenuated tumor metastases, but had no effect on the extent of angiogenesis or tumor size of the primary tumor (53, 54), suggesting that the CXCL12–CXCR4 biological axis promotes metastases independently from angiogenesis (55).

In addition, chemokines are key components of immune system response against cancer cells (56, 57). On the one hand, oncogenic driver mutations and continuous immunological pressure frequently control the expression of chemokines and their receptors, dynamically modulating the tumor immune environment, on the other, the complex and dynamic network of cytokines, chemokines, and growth factors in the tumor microenvironment (TME) promotes intracellular and intercellular communication that modulates tumor/stroma interactions, including immune responses. The TME consists of resident non-cancerous cells like stromal fibroblasts, endothelial cells, and immune cells, proteolytic enzymes, growth factors, inflammatory cytokines, and the extracellular matrix (ECM) (58). In TME paracrine and autocrine signaling by chemokines, generate complex communications between tumor cells and the TME cells (59). Chemokines facilitate interaction between cancer cells and the TME’s surrounding non-neoplastic cells, such as fibroblasts and endothelial cells. Additionally, chemokines boost neutrophil infiltration and activate tumor-associated macrophages (TAM) (60). CXCL12 in the TME could be secreted by cancer-associated fibroblasts (CAF), stimulating tumor growth directly, acting through CXCR4 and promoting invasiveness (61).

Differences in quantity, composition, functional status, location, and type of immune cells of TME, have an impact on the prognosis and therapeutic response, assuming pro-tumor or anti-tumor characteristics. The TME and the immune profile of cancers have a different behavior, not only across tumor types but also among patients with the same tumor type and in different tumor sites within the same patient (62–64). In colon cancer, chemokines in TME have direct pro-tumor effects: CCL2 targeting vascular endothelial cells *via* the Janus kinase 2 (JAK2)–STAT5 and p38 mitogen-activated protein kinase pathways, CCL3 and CCL5 can promote tumor invasion and metastasis (64). In non-small cell lung cancer, CXCR4+ tumor cells may have stem-like properties, showing a high metastatic potential and radiation resistance (65). In pancreatic cancer the CC-chemokine ligand 20 (CCL20) and its receptor CC-chemokine receptor 6 (CCR6) mediate the cancer migration and metastasizing. CCL20 is the strongest target gene in TNF-related apoptosis-inducing ligand (TRAIL) resistance through the NF-kB subunit RelA. Both autocrine CCL20 and paracrine immune cell recruitment help pancreatic cancer cells develop TRAIL resistance (66). Immune cells known as TAM that exhibit M2-type macrophage markers have been found to accelerate tumor growth and inhibit cytotoxic T-cell defenses. Additionally, IL4-stimulated M2-type macrophages highly express CCL20 and support pancreatic cancer cell invasion and the epithelial-mesenchymal transition (EMT). Another study found that the CCL20-CCR6 axis enhanced liver metastasis and pancreatic cancer growth in a mouse model (67).

Finally, The CXCL12-CXCR4-CXCR7 axis promotes tumor-related inflammation and metastasis, by regulating the trafficking of immune and tumor cells (68–70), while recent research has highlighted an immunosuppressive role of the CXCL12/CXCR4 axis through the recruitment of specific populations of immunosuppressive cells within the TME (71)

In pancreatic cancer, a high expression of CXCL12 and CXCR4 is correlated with a worse prognosis (72, 73).

### 3.2 CXCL12 role in carcinogenesis and progression: Preclinical studies and observational evidence in solid tumors, including PDAC

As described above, CXCL12 and its receptors activate key survival pathways, playing a central role in several processes involved in cancer development and growth, including PDAC (74). In solid and hematologic malignancies, the CXCL12-CXCR4-CXCR7 axis was related to resistance to therapies. Moreover, high CXCL12 expression correlates with worst survival in patients with esophagogastric, pancreatic or lung cancer (72). Indeed, CXCL12 can promote local invasion of cancer cells, while loss of CXCL12 promotes tumor cell migration to organs expressing high levels of CXCL12 such as the liver, bone marrow and lung (72).

In mycosis fungoides (MF) the CXCL12/CXCR4 axis is one of the mechanisms by which CAFs promote tumor cell migration and drug resistance. A study demonstrated that CXCL12 was significantly upregulated in fibroblasts of MF patients when compared to control, enhancing MF resistance to chemotherapy (75). Similarly, CXCL12 and its receptors CXCR4 and CXCR7 are also involved in prognosis and progression of different solid tumors (76). In breast cancer CXCR7-deficient mouse, higher local recurrences were reported after resection, suggesting that CXCR7 may have tumor-suppressor functions involved into the metastatic cascade (77).

With regards to PDAC carcinogenesis, increased CXCL12 levels were directly associated with increasing PanIN grade (78). CXCL12- CXCR4 axis plays an important role in the progression and organ specific metastatic spread (79), enhances cancer growth and restrict immune surveillance across the tumor through local autocrine and paracrine mechanisms (80).

Furthermore, in PDAC, CXCL12 induces shape changes in pancreatic cancer cells which make them resemble to migrating cells and increase cell migration *in vitro* (81) [e.g. increased expression of MMP-2, MMP-9 and uPA (82)]. To support tumor growth and expansion, CXCL12 axis can attract CXCR4-positive inflammatory, vascular, and stromal cells into the tumor mass, favoring a cross talking process with TME (83). The crosstalk between CXCL12/CXCR4 signaling and EMT and loss of cell adhesion which promotes cell cycle progression (82) was highlighted also in other type of cancers, like human sacral chondrosarcoma (84).

In PDAC tissues and pancreatic cancer cell lines SATB-1 - a nuclear matrix attachment region-binding protein which controls the transcription and expression of all gene - is overexpressed, and CXCL12, which is released by CAFs, increases SATB-1 expression (85, 86).

CXCR4 and CXCR7 are often co-expressed in PDAC specimens and cell lines, with discordant results. In early pancreatic cancer cell lines, CXCR7 knockdown, but not CXCR4 knockdown, does arrest CXCL12- mediated proliferative changes. Opposite results were obtained for the metastatic pancreatic cancer cell line. These results indicate that both CXCR4 and CXCR7 can mediate CXCL12-driven proliferation. CXCR4 and CXCR7 signaling is b-arrestin-2-dependent and controls CXCL12 signals to the MAPK pathway (87).

In advanced colorectal cancer, CXCR7 expression is associated with lymph node metastasis and progression, being employed as prediction marker for lymph node involvement (88). Generally, tumors that exploit CXCL12/CXCR4 axis tend to grow and metastasize in tissues like the liver, lung, adrenal glands, and bone marrow, whereas tumors that exploit CXCL12/CXCR7 axis are more often responsible for lymphatic metastases due to CXCR7 expression in endothelial cells, T and B lymphocytes, and dendritic cells (13, 89). The interplay among CXCL12/CXCR4/CXCR7 axis and metastases is reinforced by a preclinical study that evidenced how the systemic treatment with CXCR7 antagonists reduces tumor expansion in lungs of experimental mice inoculated with HT-29 or C26 cancer cells (90).

In small cell lung cancer (SCLC), CXCL12 induces the overexpression of CXCR4 through some integrins (2, 4, 5, and ß1 integrins), which results in a significant affinity of cancer cells for extracellular matrix and confers chemoresistance to tumors. CXCR4 inhibitors may lessen this chemoresistance effect (91).

The CXCL12-CXCR4/CXCR7 axis is a highly conserved mechanism for organogenesis and tissue healing in gastrointestinal malignancies (gastric, pancreatic, and colorectal cancer). A complete knockout is incompatible with life. Cancer cells may be able to take advantage of this chemokine pathway due to genetic abnormalities. Increased CXCR4 expression on tumor cells not only enable them to spread to CXCL12 gradients that are phisiologically present in the body, but CXCR4 binding on tumor cells also promotes resistance to therapies by reducing pro-apoptotic signaling. Additionally, by interfering with proper immune cell migration, changes in the ability of TME stromal cells to produce CXCL12 may hinder effective cell-mediated tumor killing, resulting in enhanced tumor growth, showing how CXCL12-CXCR4/CXCR7 axis could be considered as a mechanism of immune resistance in gastrointestinal malignancies (92).

Considering CXCL12–CXCR4/CXCR7 axis involvement in survival, tumor growth, angiogenesis, metastasis, TME, and drug resistance, it has been investigated as promising target for therapeutic interventions (93, 94).

### 3.3 -The axis CXCL12-CXCR4-CXCR7 in PDAC: Could it be considered as new potential biomarkers and therapeutic targets?

CXCL12-CXCR4-CXCR7 axis has been associated to cancer progression in several tumor types (53, 95, 96). Thus, it seems interesting to investigate their possible role in PDAC environment. The TME induces changes in cellular components (97) by secreting exosomes and soluble factors such as VEGF, CXCL1, and CXCL8- (98, 99) to promote colonization (100, 101). As previously described, in PDAC, TME may have a tumor-promoting or tumor-suppressive action based on the expression of specific factors such as CAFs, stellate cells, different immune cells and cytokines (102). Hence, the TME, with its complexity of signal pathways (103), acts as a key determinant by which PDAC acquires therapeutic resistance to currently available treatment and it could represent a target for new therapeutic strategies based on context-dependent stromal alterations (104, 105). CXCL12/CXCR4 axis is one of the most prominent chemokine moderators of the TME (68). The examination of formalin-fixed paraffin embedded (FFPE) specimens obtained from patients who had undergone resection for pancreatic adenocarcinoma, showed both high frequency of CXCR4/CXCR7 co-expression in human pancreatic cancer tissues compared to normal tissues and the increase in staining intensity over tumor stage (87), confirming a decisive role in the pathogenesis of PDAC.

In literature has been reported the effect of upregulation of SATB-1 – a nuclear matrix attachment region-binding protein called AT-rich sequence-binding protein 1- by CXCL12, CAFs of pancreatic cancer stroma *via* the CXCL12/CXCR4 axis. CXCL12/CXCR4/SATB-1 axis accounts for the malignant progression, but also gemcitabine resistance of pancreatic cancer cells, suggesting a potential target of chemosensitivity in therapeutic management of PDAC patients (86). In addition, CXCL12 overexpression correlated with the lymph node metastasis, whereas SATB-1 overexpression correlated with differentiation, T stage, TNM stage, and lymph node metastasis. Furthermore, higher CXCL12 or SATB-1 staining intensity correlated with poorer prognosis in PDAC patients suggesting also a possible prognostic role in these tumors (86).

The implication of chemokines in PDAC tumorigenesis, prognosis, and resistance to therapy and the increasingly urgent need to understand the signaling mechanisms, supported the development of molecularly targeted pancreatic cancer therapies.

Recently, several preclinical and ongoing clinical studies have been analyzing the therapeutic option of CXCL12-CXCR4/CXCR7 axis. Moreover, different molecules have been developed in several areas of medicine as antagonists of the CXCR4 receptor (106, 107). *In vitro* studies showed that, resistance to gemcitabine mediated by CXCR4 chemokine receptor (108) and polo-like kinase 1 (PLK1) (109, 110) can be overcome combining a polymeric cholesterol-modified CXCR4 (PAMD-CHOL) antagonist to a PLK1 knockdown by siRNA. The biodistribution of the nanoparticles in orthotopic pancreatic cancer models revealed strong accumulation in primary and metastatic tumors. In a therapeutic study *in vivo*, the triple combination of gemcitabine with PAMD-CHOL/siPLK1 showed superior anticancer activity when compared with single and dual combination controls (111). An interesting retrospective analysis of the INT-11 trial demonstrated that tipifarnib, a farnesyl transferase inhibitor that inhibits CXCL12 expression in pancreatic fibroblasts, could sensitize PDAC to gemcitabine in a subset of PDAC patients (112).

In orthotopic pancreatic tumor–bearing mice treated with gemcitabine combined to a CXCR4 antagonist (AMD3100) or hedgehog inhibitor (GDC-0449) showed significant suppression of tumor growth. Those target agents act on a pathway signaling which promote pancreatic tumor desmoplasia by inducing proliferation and differentiation of pancreatic stellate cells into myofibroblasts (113).

The involvement of CXCR4 in radiation resistance was previously demonstrated in several tumors (114–116). Kato and colleagues confirmed the correlation between the high intensity immunostaining of CXCR4 in pancreatic cancer cells and survival or stage of cancer. Moreover, they found that CXCR4 expression and invasion were enhanced in radiation-resistant pancreatic cancer cell lines compared to normal cancer cell lines. The CXCR4 antagonist AMD070 suppressed the cancer cell invasion enhanced by CXCL12 treatment, and when used in combination with irradiation, AMD070 suppressed the colonization of radiation-resistant pancreatic cancer cells (117). Alternatively, CXCR4-CXCL12 axis can be inhibited by directly preventing CXCL12 binding to CXCR4 or CXCR7. Olaptesed pegol (NOX-A12) is a novel non-immunogenic synthetic oligonucleotide that inhibits the chemoattractant stromal cell-derived factor-1 (SDF-1 or CXCL12) (118, 119). Currently, there is a Phase 1/2 clinical trial looking at Olaptesed in combination therapy with Pembrolizumab in metastatic pancreatic and colorectal cancers (NCT03168139). The preclinical studies focused on the therapeutic role of CXCL12-CXCR4/CXCR7 axis are summarized in **Table 1**.

**Table 1:** Preclinical studies about therapeutic option of CXCL12-CXCR4/CXCR7 axis in PDAC.

Experimental Treatment	Type of study (vivo/vitro)	Disease Involved in the trial	Mechanism of action	Biological effect mediated by experimental treatment
**Evaluation of CXCR4 mRNA (PaCA cells) and CXCL12 mRNA (fibroblasts) by RT-PCR** **Expression of CXCR4 protein (PaCa cells) by ELISA** (108)	Vitro	Pancreatic Cancer	–	GEM resistance: CXCR4 in GEM-R PaCa cells was significantly enhanced by GEM but not in normal GEM-sensitive (GEM-S) PaCa cells. In RT-PCR and ELISA assays, the production and secretion of CXCL12 from fibroblasts was significantly enhanced by co-culturing with GEM-R PaCa cells treated with GEM
**AMD11070 and KRH3955** (108)	Vivo (mice)	Pancreatic Cancer	Effects of CXCR4 antagonists (using Matrigel invasion assays and animal studies) on the invasiveness and tumorigenicity of GEM-R PaCa cells stimulated by CXCL12	In Matrigel invasion studies, fibroblast-derived CXCL12 increased the invasiveness of GEM-R PaCa cells treated with GEM, while AMD11070 and KRH3955 significantly decreased it. GEM enhanced the tumorigenicity of GEM-R PaCa cells *in vivo*, whereas the addition of CXCR4 antagonists dramatically reduced it.
**Exposure of fine-needle aspiration material of PDAC to control vehicle or gemcitabine** (109)	Vitro	Pancreatic cancer	6 h exposed fine-needle aspiration material of PDAC to control vehicle or GEM (1 mumol/L) and compared the gene expression of the treated and untreated samples using a reverse transcription-PCR-based, customized low-density array with 45 target genes of therapeutic interest.	Plk1 gene, which showed >50% downregulation in sensitive cases vs resistant.
(109)	Vivo	Pancreatic cancer	–	In GEM-R pancreatic cancer xenografts showed synergistic activity decreasing cell proliferation and tumor regressions
**Injection of nanoparticles with cholesterol modified PAMD and siPLK1 plus gemcitabine treatment *in vitro* in both murine and human pancreatic cancer cell lines** (111)	Vitro	Pancreatic cancer	Combining CXCR4 inhibition by a polymeric CXCR4 antagonist PAMD-CHOL with PLK1 knockdown by siRNA, enhance the therapeutic effect of gemcitabine in orthotopic model of metastatic pancreatic cancer	Biodistribution of the nanoparticles in orthotopic pancreatic cancer models revealed strong accumulation in primary and metastatic tumors. The cholesterol-containing nanoparticles showed not only increased tumor accumulation than the cholesterol-lacking control but also deeper penetration to the tumor
(111)	Vivo	Pancreatic cancer	–	Triple combination of PAMD-CHOL/siPLK1 and gemcitabine showed superior anticancer activity when compared with single and dual combination controls
**CXCR4 antagonist (AMD3100) or hedgehog inhibitor (GDC-0449)** (113)	Vitro	Pancreatic cancer	The monocultures and co-cultures treated with gemcitabine in the presence or absence of AMD3100 and/or GDC-0449 and measured the viability of PC cells	PaCa cells co-cultured with pancreatic stellate cells (PSCs) are significantly more resistant to gemcitabine toxicity than those grown in monoculture. The co-culture–induced chemoresistance is abrogated by inhibition of the CXCR4 and hedgehog pathways
(113)	Vivo (mice)	Pancreatic cancer	CXCR4 antagonist (AMD3100) or hedgehog inhibitor (GDC-0449	Treatment of orthotopic pancreatic tumor–bearing mice with gemcitabine alone or in combination with AMD3100 plus GDC-0449 displays reduced tumor growth. The triple combination treatment is the most effective (Immunohistochemical analysis of Ki67 and cleaved caspase-3 confirm these findings from *in vivo* imaging and tumor measurements)
**The role of the CXCL12/CXCR4 axis in radiation resistance in PaCa** (117)	Vitro	Pancreatic cancer	Involving of CXCL12/CXCR4 axis in the radiation resistance of PaCa	The expression of CXCR4 was higher in radiation-resistant PaCa cell lines than in normal PaCa cell lines. The invasion ability of radiation-resistant PaCa cell lines was greater than normal cell lines and was enhanced by CXCL12 treatment
**AMD070** (117)	Vitro	Pancreatic cancer	The effects of a CXCR4 antagonist on radiation resistant PaCa cell lines	Suppression by the CXCR4 antagonist AMD070 of the enhanced invasion ability of PaCa cells
**Olaptesed pegol (NOX-A12)** (118)	Vivo (phase II)	Pancreatic and colorectal cancers	NOX-A12 inhibits the chemoattractant stromal cell-derived factor-1 (SDF-1 or CXCL12)	–

RT-PCR, reverse transcriptase-polymerase chain reaction; ELISA, enzyme-linked immunosorbent assay; PaCa, Pancreatic Cancer; GEM, Gemcitabine; GEM-R, Gemcitabine resistant; GEM-S, Gemcitabine sensible; PDAC, Pancreatic Ductal Adenocarcinoma; PAMD-CHOL, CXCR4-inhibiting polymer; PSCs, pancreatic stellate cells; siPLK1, siRNA-mediated polo-like kinase 1.

### 3.4 Immunoresistance phenotype of PDAC: The role of immunotherapy

The TME of PDAC is highly immunosuppressive and is characterized by an extensive and dense fibrous stroma that can account for up to 80% of the total tumor volume (120) and is responsible for an abundant desmoplastic reaction, creating a physical barrier that makes the drug perfusion difficult (121). The crosstalk between pancreatic cancer-associated stroma, cancer cells and soluble proteins such as cytokines and growth factors (122) could represent a target of therapeutic strategies modulating tumor-promoting and tumor-suppressive signals (104, 105). The immune resistance of PDAC cannot be explained only by the presence of desmoplastic peritumoral reaction, but also by further mechanisms such as the minimal antitumor T cells infiltration (123) and the synergism with other regulatory immune cells to evade immune surveillance (124), tumor cell-intrinsic pathways involving the KRAS oncogene (125) and its downstream effector PIK3CA (126), the tumor suppressor gene p53 (127) and NF-κB that contributes to tumor growth by increasing the expression of CXCL12, which prevents cytotoxic T cells from infiltrating the tumor and killing cancer cells (128, 129).

The production of immunosuppressive chemokines and cytokines represents one important mechanism of immune evasion in PDAC and the CXCL12-CXCR4/CXCR7 chemokine axis, as described above, has a prominent role in modulating the trafficking of immune cell populations in the microenvironment (92).

Although all these evidences highlights how the TME may represent an area of interest in pancreatic cancer immuno-therapeutic exploration, the efficacy of immunotherapy in PDAC appeared poorly encouraging survival benefit (130–132) despite the observation of PD1 expression in such patients (130, 133, 134). Indeed, the PDAC was historically defined as a non-immunogenic tumor that displays a paucity of antigens to be recognized as foreign by the host immune system, the presence of peripheral T cells specific for an abundant PDAC antigen, mesothelin, and the lack of these T cells in the tumor microenvironment are consistent with the existence of local immunosuppression (135). On the one hand the limited T-cell infiltration has been hypothesized to be one crucial reason for the failure of checkpoint inhibitor therapy (136–138), on the other, the inhibition of the CXCL12/CXCR4 axis appeared as a promising TME modulatory strategy to improve ICIs outcomes (136, 139).

Considering the mechanism of immunomodulation of the CXCL12-CXCR4-CXCR7 axis, and of the potential interaction with the microenvironment in the PDAC, several combined therapeutic approaches have been studied and developed, to overcome the “cold” immunological setting of PDAC.

Recently, a dose escalation trial was conducted with the objective to assess the impact on the tumor microenvironment of selective CXCR4 antagonist named AMD3100 - also known as plerixafor - in advanced PDAC, ovarian and colorectal cancer, to identify the proof of mechanism, by demonstrating alterations in T-cell tumor distribution, ideally associated with loss of tumor cells, measured by immunostaining, and changes in FDG uptake (NCT03277209).

The depletion of tumor stroma or targeting CXCL12 from CAF exhibited a synergistic effect with immune checkpoint inhibitors in PDAC generating genetically engineered mouse models, suggesting that the conditioning of the PDAC stroma could be a prerequisite for attaining therapeutic responses to immunotherapies (136, 140). An interesting preclinical study showed how in a murine pancreatic cancer model, despite the presence of antitumor T cells, immunotherapeutic antibodies (anti-PDL1) are ineffective, but removing the CAF expressing fibroblast activation protein (FAP) from tumors or inhibiting the interaction of its chemokine, CXCL12, with CXCR4, permitted immune control of tumor growth and uncovered the efficacy of these immunotherapeutic antibodies. FAP+ CAFs are the only tumoral source of CXCL12 and administering AMD3100 also revealed the antitumor effects of an immunotherapeutic antibody and greatly diminished cancer cells (136).

Inhibiting CXCR4 by continuous infusion of AMD3100 in an experimental microsatellite stable (MSS) PDAC and colorectal cancer human cells, induced an integrated immune response with intratumoral T and NK cells accumulation and activation and B cell response, that was detected by transcriptional analysis of paired biopsies of metastases from patients (141). Combination therapy showed efficacy through intravenous injection of traps - plasmid DNA encoding for CXCL12 and PD-L1 - in mice bearing orthotopic pancreatic cancer. Expression of traps was mainly seen in the tumor. Moreover, combination trap therapy shrunk both tumor and metastases, and significantly prolonged the host survival. The authors also found a modification of the immunosuppressive TME as CXCL12 trap allowed T-cell penetration into the tumor, and PD-L1 trap allowed the infiltrated T-cells to kill the tumor cells (139).

In clinical practice most studies addressing the CXCL12-CXCR4 axis have tackled hematological malignancies (142, 143), while in solid tumors the trials are less numerous (**Table 2**).

**Table 2:** Recruiting and completed trials of CXCL12-CXCR4-CXCR77 inhibitors plus immunotherapy in PDAC.

Trial Name	Disease Involved in the trial	Number patients enrolled	Experimental Treatment/Control	Line of Therapy	Phase	Primary EP	mOS (mo)	mPFS (mo)
***NCT03277209***	**Advanced pancreatic, high grade serous ovarian or colorectal adenocarcinoma**	2	AMD3100 (Plerixafor)	–	I	Causality of AEs and SAEs and grading according to NCI CTCAE v.4.03	–	–
***NCT02737072*** (144)	**PDAC (8 patients), colorectal (1 patient)**	9	LY2510924 + Durvalumab	–	Ia/Ib	Number of Participants Who Experienced DLTs, MTD of LY2510924	–	–
***COMBAT*** (145)	**Metastatic PDAC**	37	BL-8040 (motixafortide) + Pembrolizumab and chemotherapy	≥ 2	IIa	ORR	3.3	–
***KEYNOTE-559/OPERA*** (146)	**MSS Colorectal Cancer (11 patients) or MSS pancreatic cancer (9 patients)**	20	Olaptesed (NOX-A12) +/- Pembrolizumab	≥ 2	I/II	Evaluation of changes within the tumor microenvironment induced by CXCL12 inhibition with NOX-A12 by comparing pre- and post-treatment biopsy specimens. Safety and tolerability of NOX-A12 plus pembrolizumab.	3.97	1.87
***NCT04177810***	**Pancreatic cancer**	Recruiting	AMD3100 (Plerixafor) + Cemiplimab	>1	2	Objective Response rate, G3 or above toxicities		

mOS, median overall survival; mPFS, median progression free survival; mo, months; EP, endpoint; AEs, adverse events; SAEs, severe adverse events; DLTs, dose-limiting toxicities; MTD, maximum tolerated dose; ORR, objective response rate.

The attractive immunomodulating role of CXCR4 was investigated in phase I in which the primary objective was to assess the maximum dose tolerated and safety of an antagonist of CXCR4 LY2510924 administered subcutaneous daily in combination with durvalumab in patients with advanced solid tumors (8 PDAC, 1 colorectal). No dose limiting toxicities were reported and about activity four patients reported stable disease and one patient partial response (144).

In pretreated PDAC patients, monotherapy with BL-8040 - an inhibitor of CXCR4 activity - followed by repeated cycles of pembrolizumab every 3 weeks and BL-8040 three times weekly, showed a DCR of 34.5%, although only one patient reported a partial response. After this priming phase and based on the results of this initial cohort, the expansion cohort of triple combination therapy of BL-8040, pembrolizumab and irinotecan-based chemotherapy, showed surprisingly, an ORR, DCR and median duration of response of 32%, 77% and 7.8 months respectively, suggesting a possible expansion of the chemotherapy’s benefit by combined CXCR4 and PD-1 blockade (145).

In the phase I/II Keynote-559 patients affected by microsatellite stable (MSS) metastatic colorectal and pancreatic cancers were treated with a fixed dose of olaptesed pegol (also known as NOX-A12) as a monotherapy for 2 weeks, followed by a combined therapy with 200 mg pembrolizumab one time every 3 weeks until disease progression or limiting toxicity. Olaptesed pegol is an oligonucleotide that binds to SDF-1 thereby preventing the binding of SDF-1 to its receptors CXCR4 and CXCR7 and blocking the subsequent receptor activation, with consequent prevention of angiogenesis, proliferation, invasion and metastasis. Furthermore NOX-A12 facilitating the influx of immune effector cells into solid tumors, may allow an effective response to immune checkpoint inhibitors. The treatment with NOX-A12 and pembrolizumab was well tolerated and demonstrated to be able to stabilize the disease in heavily pretreated MSS patients for prolonged periods, for almost a quarter of the patient’s overall survival close to 12 months could be reached (146).

## 4 Future perspective and conclusions

Considering the critical and non-systematic nature of this review, and the heterogeneity of the included studies, which not allow conclusive evidence, our work highlights the crucial role that plays CXCL12-CXCR4-CXCR7 axis in carcinogenesis, disease progression and drug resistance of pancreatic cancer. Moreover, compared to previous published reviews (80, 147), ours work focused on the immunomodulatory activity of this chemokine’s axis, speculating on the possibility of exploiting them for therapeutic purposes.

This axis represents, as reported in a recent meta-analysis a potential predictive factor of worse prognosis of PDAC, esophagogastric and lung cancers (72). Consequently, a deeper understanding of the pancreatic cancer biology, including new insight into the tumor metabolism and TME, could lead to the development of promising and innovative therapeutic strategies.

According to this evidence, several preclinical and clinical studies are ongoing to analyze therapeutic opportunities targeting the CXCL12-CXCR4/CXCR7 axis in the complex field of pancreatic TME. Currently it has been widely supposed that targeting a single molecule or single pathway was unlikely to yield more pancreatic cancer therapies, therefore future approaches about combination and/or multi-modal strategies that target multiple features of the TME simultaneously might be more successful than the single agent therapy. Furthermore, while the possibility to overcome resistance to radiotherapy and chemotherapy treatment has been already investigated - although mainly in preclinical studies - the immunomodulating role of CXCL12-CXCR4/CXCR7 antagonists could represent a future challenge in the therapeutic landscape of PDAC.

In conclusion, the present review suggests how modulating TME with a combination of chemotherapy, PD-1/PD-L1 and CXCL12-CXCR4-CXCR7 axis antagonists could represent one of the future therapeutic options to improve the immune responses in immune refractory cancers, including PDAC.

## Author contributions

Conception/Design: MR. Data analysis and interpretation: MR, GA. Manuscript writing: GA, MC, MR. Final approval of manuscript: MR, GA. Supervision: GB, PM, FM, EP, DS. All authors contributed to the article and approved the submitted version.
